# Organic geochemical evidence of human-controlled fires at Acheulean site of Valdocarros II (Spain, 245 kya)

**DOI:** 10.1038/s41598-023-32673-7

**Published:** 2023-05-18

**Authors:** Lavinia M. Stancampiano, Susana Rubio-Jara, Joaquín Panera, David Uribelarrea, Alfredo Pérez-González, Clayton R. Magill

**Affiliations:** 1 Lyell Centre for Earth & Marine Science & Technology, Heriot-Watt University, Edinburgh, UK; 2 Centro Nacional de Investigación Sobre la Evolución Humana (CENIEH), Burgos, Spain; 3 Instituto de Evolución en África (IDEA), Madrid, Spain; 4 Universidad Complutense de Madrid, Madrid, Spain

**Keywords:** Biogeochemistry, Biogeochemistry, Environmental sciences

## Abstract

Among the outstanding questions about the emergence of human-controlled fire is the systematic recurrence between the geochemical remains of fire and its preservation in the archaeological record, as the use of fire is considered a technological landmark, especially for its importance in food cooking, defensive strategies, and heating. Here we report fossil lipid biomarkers associated with incomplete combustion of organic matter at the Valdocarros II site, one of the largest European Acheulean sites in Spain dated to marine isotopic stage (MIS) 8/7 (~ 245 kya) allowing a multiproxy analysis of human-controlled fire use. Our results reveal isolated cases of highly concentrated and diverse polycyclic aromatic hydrocarbons (PAHs) and alkylated PAHs (APAHs), along with diagnostic conifer-derived triterpenoids in two hearth-like archaeological structures. The presence of combustion byproducts suggests the presence of anthropogenic (controlled) fires at Valdocarros—one of the oldest evidence of fire use in Europe-in association with Acheulean tools and bones. Hominins possibly used fire for two main activities, as a means of defense against predators and cooking. Our results help to better delineate major gaps in our current knowledge of human-controlled fire in the context of the Middle-Pleistocene in Europe and suggest that human ancestors were able to control fire before at least 250 kya.

## Introduction

Controlled fire is a technological milestone in the history of human evolution. Our understanding of fire control has a direct impact on what it means to be ‘human’. It distinguished our species from other animals because it led us spread to much colder regions, develop powerful defensive implements, and increased caloric intake vis-à-vis cooked food^1,2^*. *In situ archaeological hearths—which can include charcoal, fire-altered biomass or sediment, or charred features on artifacts—provide direct evidence of human-controlled fire. Usually, archeological sites located in caves better preserve artifacts and hearths compared to open-air sites, which are exposed to meteorological conditions that could erode the burning evidence. However, the study of fire amid human evolution remains controversial because of the difficulties of identifying potential hearth residues in the archeological record due to diagenesis and spatiotemporal reworking^1^.

### Past records of fire within human evolution

**Figure 1:**
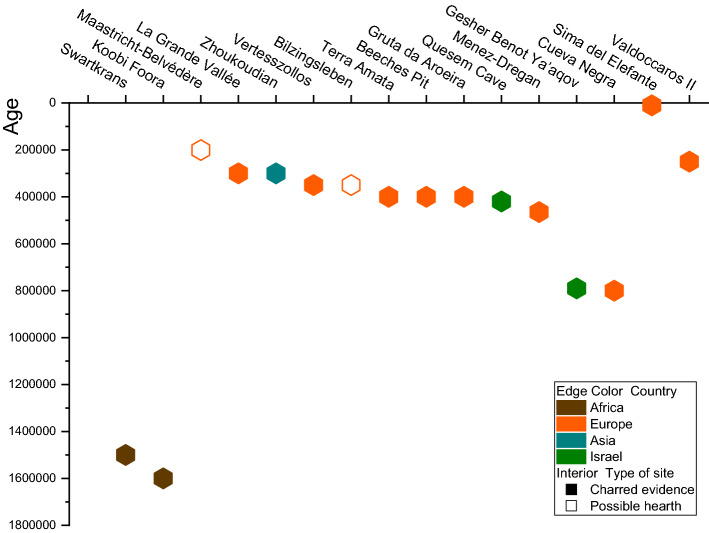
Timeline showing some of the most informative fire sites from Europe to Asia. The color-coding shows approximate the continent/country and the time extent of the archeological site.

The oldest evidence that relates hominins with fires was discovered in Africa with an estimated age of 1.5 Ma in the Swartkrans cave (Fig. 1), South Africa, where 270 burnt bones were registered^3^. In GnJi 1/6 in Chesowanja 1.42 Ma, thermo-altered clays were identified, and at FxJj 20 Main in Koobi Foora (ca. 1.6 Ma) in Kenya, which contains oxidized sediments. Yet, the controlled use of fire at these locations has been widely doubted^4^. Recently, at FxJj 20 AB in Koobi Foora, Kenya, (ca. 1.5 Ma), a study reported evidence of thermally altered lithics, sediments, and bone fragments using FTIR analyses^5^. Another problematic site is 8E Gadeb (1.45–0.7 Ma) in Ethiopia, where stones with signs of heat alterations were registered^6^. Later, ca. 1 Ma, at Wonderwerk cave (South Africa) evidence of fire is indicated by the presence of ashed plant remains, and burnt bones associated with Acheulean tools^7^.

Outside of Africa, the earliest clear evidence of anthropogenic fires has been recorded in the Near East. In Israel, at the open-air Acheulean site of Gesher Benot Ya’aqov (790 ka) charred plants and thermally altered lithics were recorded in several levels^8^. At Quesem Cave, which dated to 420–200 ka, wood ash was identified deriving from a fireplace associated with burnt bones and burnt lithics^9^, and in Tabun Cave dated to 357–324 ka were recorded numerous burnt flints^10^.

In Europe, between MIS 13 and MIS 9, it is widely accepted that fire was continuously used^11,12^, and some authors link the control of fire with the expansion of Acheulean technology in Europe, ca. 500–600 ka^13^. Several sites with different evidence have been described: Vertesszollos (Hungary) close to 350 ka possible hearths and burnt bones^14^; Menez-Dregan 1 (France) with fireplaces associated with charcoals and burnt tools (around 465 and 380 ka)^15,16^; La Grande Vallée (France) with burnt chert^17^; Terra Amata (France) charcoal and burnt material^18^; Bilzingsleben (Germany) dated between 350–320 ka and 414–280 ka, with accumulations of burnt remains forming semi-circular areas^19^; and Schöningen (Germany) with possible hearths, burned sediments and wood; Beeches Pit (England) dated to MIS 11 with burnt flints, bones and thermally altered sediments in areas of ca. 1 m^2^^20^, and Gruta da Aroeira (Portugal), dated to ca. 400 ka, with by-products of burning^21^.

From about MIS 9, extensive evidence of fire-use has been described in open air and cave settings^22^. In east Asia at Zhoukoudian, level 4 dated 292–312 ka (TL) contains some evidence for in situ use of fire^23^; Maastricht-Belvédère (The Netherlands), dated to 250 ka and associated with Middle Paleolithic industry^24^ concentrated several heat-altered fragments in two groups which suggested a potential structure for open-air combustion, although Roebroeks^25^ proposes a natural origin for such concentrations; at La Cotte de Saint Brelade (Jersey), with a chronology, ca. 230 ka, high densities of burned bone were present in layers C and D though no evidence of hearths^26^ with a lithic assemblage associated to Late Middle Paleolithic^26^.

In the Iberian Peninsula, before MIS 13, indirect evidence of anthropogenic fires was found at level TE19 G at the Sima del Elefante site (Atapuerca, Burgos), with a chronology of less than 780 Ma, and it consisted of dispersed fragments of charcoal and absent lithic industry^27^. At Cueva Negra del Rio Quípar (Murcia), dated ca. 990–772 ka, were found thermally altered bone and heat-shattered chert flakes and cores with only one handaxe^28–30^. The Middle Pleistocene Gruta da Aroeira (Portugal) recorded human-controlled fire, dated to ca. 400 ka, in association with the Acheulean Technocomplex^21^. The Middle-Late Pleistocene Bolomor Cave (Valencia) documented reiterative use of fire due to the presence of hearths at levels II, IV, XI, and XIII, in this level the hearths dated to MIS 7c (228 ± 53 ka by AAR)^31^. Such burned remains and thermo-altered sedimentary deposits were in the primary position and the lithic technology is framed within of Ancient Middle Paleolithic, no Acheulean technocomplex^31^. At the Abrigo de la Quebrada site (Valencia), levels with Middle Palaeolithic industry, dated between 40,500 and 83,200 B.P., were registered a high percentage of wood charcoal fragments^32^.

### Valdocarros II site

**Figure 2:**
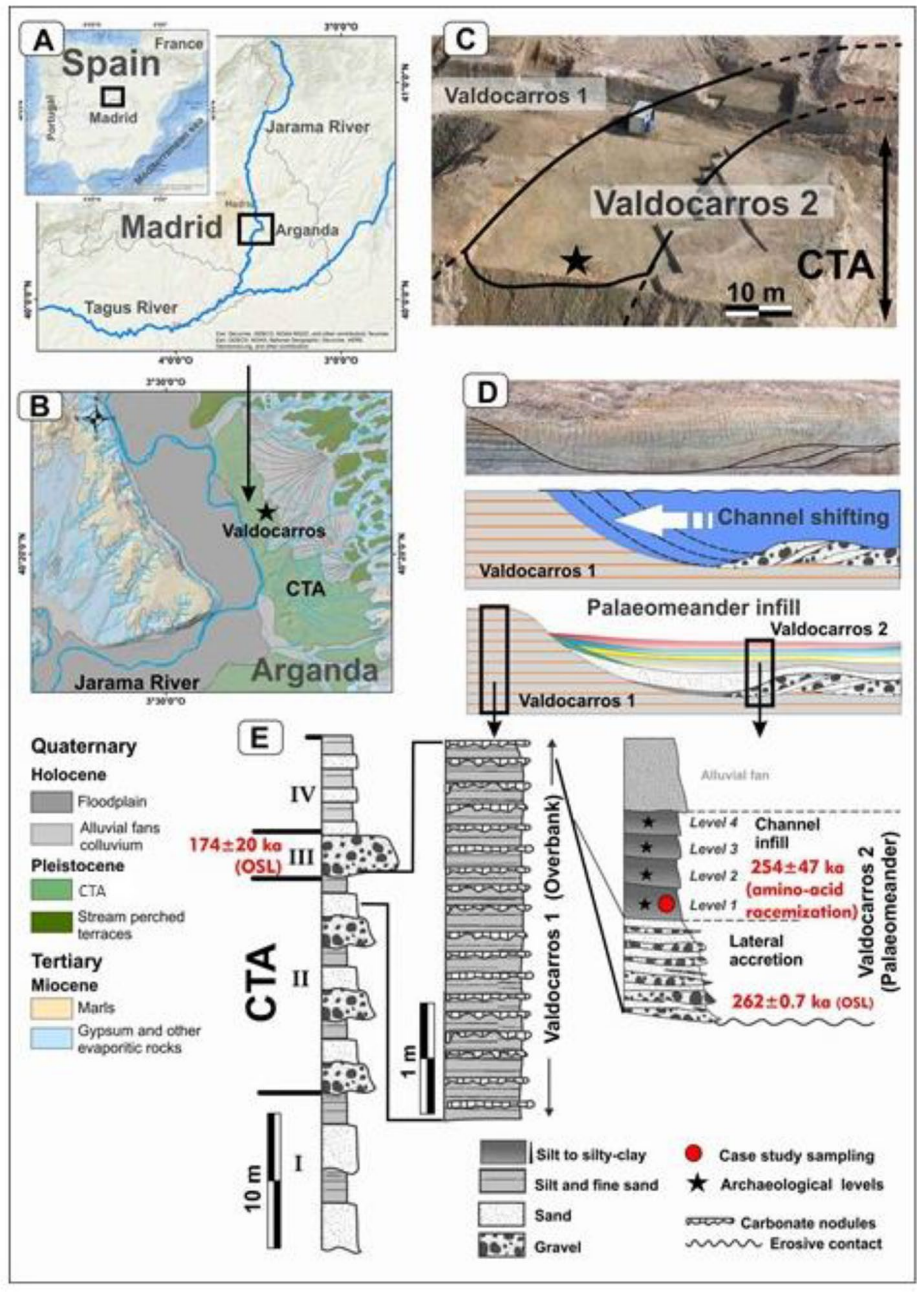
Study area and sample location. (**A**) Geographical location of Valdocarros in Spain, Madrid. (**B**) Lithology and geomorphology. (**C**) Aerial view of the gravel pit in the Complex Terrace of Arganda (CTA) and Valdocarros 2 site. (**D**) Evolution of Valdocarros 2 palaeomeander in previous overbank units. (**E**) Stratigraphy, chronology, and sampling. This figure was made using ArcGis 10.6.1. https://www.esri.com/fr-fr/arcgis/products/arcgis-desktop/resources by David Uribelarrea.

The site of Valdocarros II (Madrid, Spain, Fig. 2A,B), is located in the stratigraphical unit II of the Complex Terrace of Arganda in the Jarama river valley in the Tagus basin, with evidence of human occupancy dating to a range between 235 and 285 kya (MIS 7/8)^33–35^ and the amino-acid racemization (AAR) 254 ± 47 ka, 262 ± 07 ka) ages^33–35^ (Fig. 2E). The site is one of the very few Middle Pleistocene localities to have documented associations of bones and Acheulean stone tools in different levels^36^. A sequence of 19 terraces was previously identified in this valley^37^. These terraces, which are of stepped and perched types upstream, overlap with the oldest ones, giving rise to the Complex Terrace of Arganda (hereafter CTA) (Fig. 2B) over which the current floodplain is set^35,37,38^. The CTA is made up of successively stacked fluvial sequences, named from bottom to top Arganda I, II, and III (Fig. 2E), matching terraces + 30–32 m, + 23–24 m and + 18–20 m, respectively^35,39^. The site is one of the very few Middle Pleistocene localities to have documented associations of bones and Acheulean stone tools in different levels^36^.

Geologic evidence at Valdocarros II shows that it was located in an abandoned meander that eroded the previous overbank units (Valdocarros 1 or I) corresponding to hundreds of meters wide floodplain (Fig. 2C). The palaeomeander stratigraphy (Valdocarros 2 or II, Fig. 2D)) consists of bedload deposits (point-bar) infilled with four low-energy fluvial units of silt and clay each of 30–50 cm thick. Each layer of Valdocarros II buries an Acheulean archaeological level designated from bottom to top 1, 2, 3, and 4, respectively (Fig. 2E), demonstrating that Valdocarros II was occupied at least four times. The small grain size of its sediments and the lack of erosion structures indicate a very low-energy environment. Level 2, which covers the combustion structure has a silty loam texture composed of 26% clay, 33% silt, 25% very fine sand, 6% fine sand and 8% medium sand, hence it was formed by decantation in calm waters. Hence, the association of micromammals reinforces also the low-energy environment. Hominins collected vertebrate bones and carcasses, as well as lithic tools and raw materials to create new ones, and parts of large mammal carcasses were processed with such tools^40^. The Acheulean industry is characterized by the presence of handaxes, cleavers on flakes, and trihedral picks, made of flint and quartzite generally^36^. The floodplain concentrates hydric and biotic resources, and within it, the depression formed by the abandoned meander (Fig. 2D) and its gallery forest offer extra concealment. Groups of humans come back repeatedly to the same place, probably attracted by the vicinity to a river that provides biotic and abiotic resources and the protection of meander depression shelter unique in the landscape^36^.

Previous work in the Jarama Basin focussing on Valdocarros time suggests that climate and vegetation changes documented in that period correlate with modern climate and vegetation characteristic of the central Iberian Peninsula Meseta^41,42^. According to the herpetofauna of the site, the climate during Valdocarros was an oceanic climate through cool periods and a Mediterranean climate through the warm periods, with an of + 3 °C and – 1 °C respectively, compared to the present^43^. The malacofauna recovered from the site were moderately cold tolerant and highly tolerant of semiarid conditions^41^, but the ecologic context of this environment remains unconstrained.

**Figure 3:**
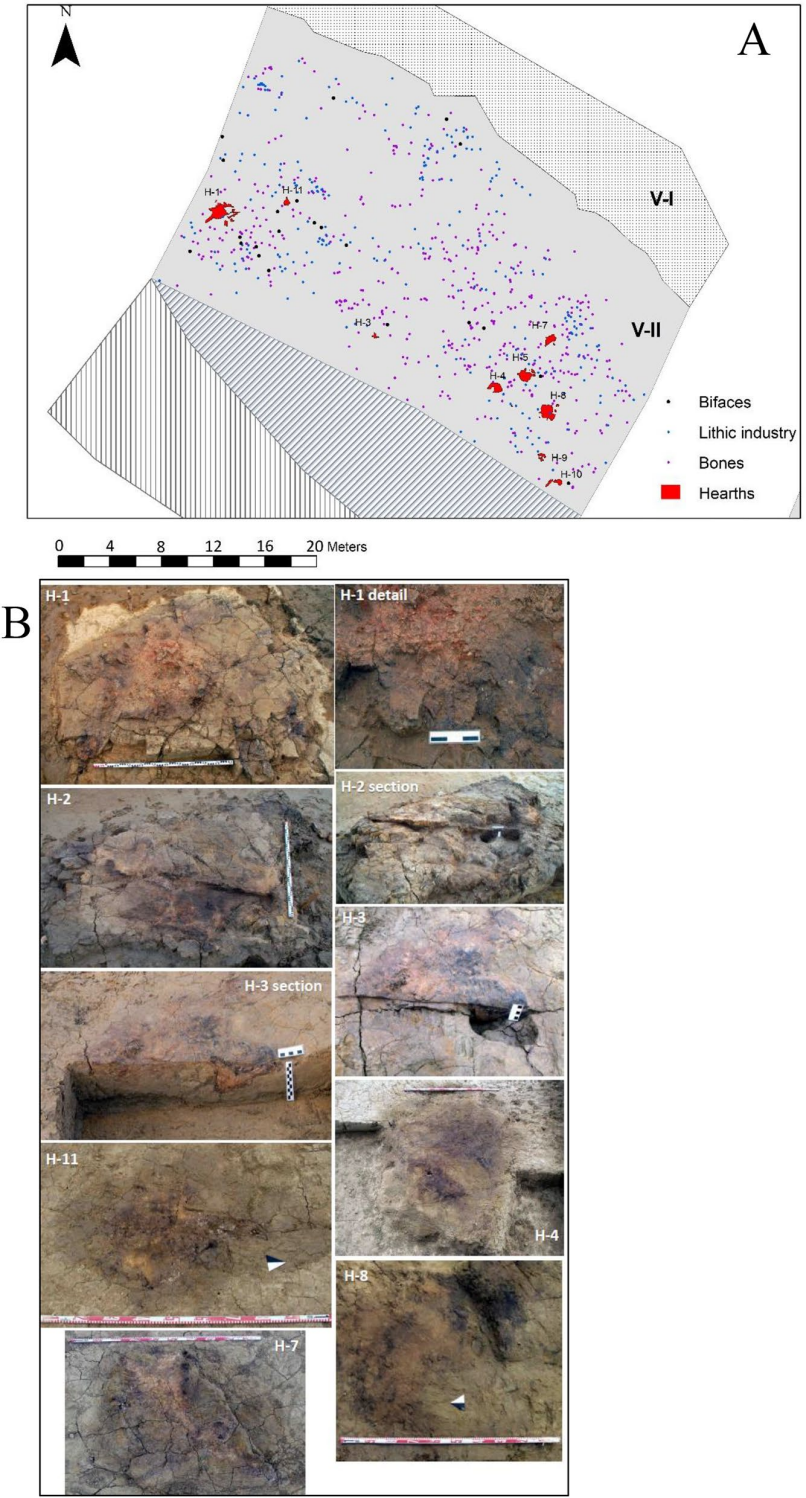
Samples location and hearths at Valdocarros II (V-II). (**A**) map of Valdocarros II Sedimentary layer 1 and spatial distribution of lithic industry, bifaces and bones. (**B**) photos taken during fieldwork the individual hearths (1,2,3,4,11,7,8). (Photos and Spatial distribution were made by Joaquìn Panera and Susana Rubio-Jara).

We report here a combined lipid biomarkers analysis of hearths sediments from level 1 (Figs. 2E, 3A,B) mostly and level 2, as well as 5 representative samples from each fluvial unit at Valdocarros II. Together with existing climatic reconstructions around the Jarama Basin and analysis of the Acheulean technology, Valdocarros II provides new insight into the emerging of human-controlled fire use and patterns of hominin local land use and behavioral dynamics in the context of the Middle Pleistocene. Our analysis reveals the presence of combustion (burning) by-products suggesting the presence of anthropogenic (controlled) fires at Valdocarros II (Fig. 3B)—some of the oldest evidence of fire-use in Europe. The burnt material consists of wood and charcoal. These finds were made in the abandoned meander and two separate occupation horizons in association with bones and Acheulean tools.

## Results and discussion

### n-Alkanes

**Figure 4:**
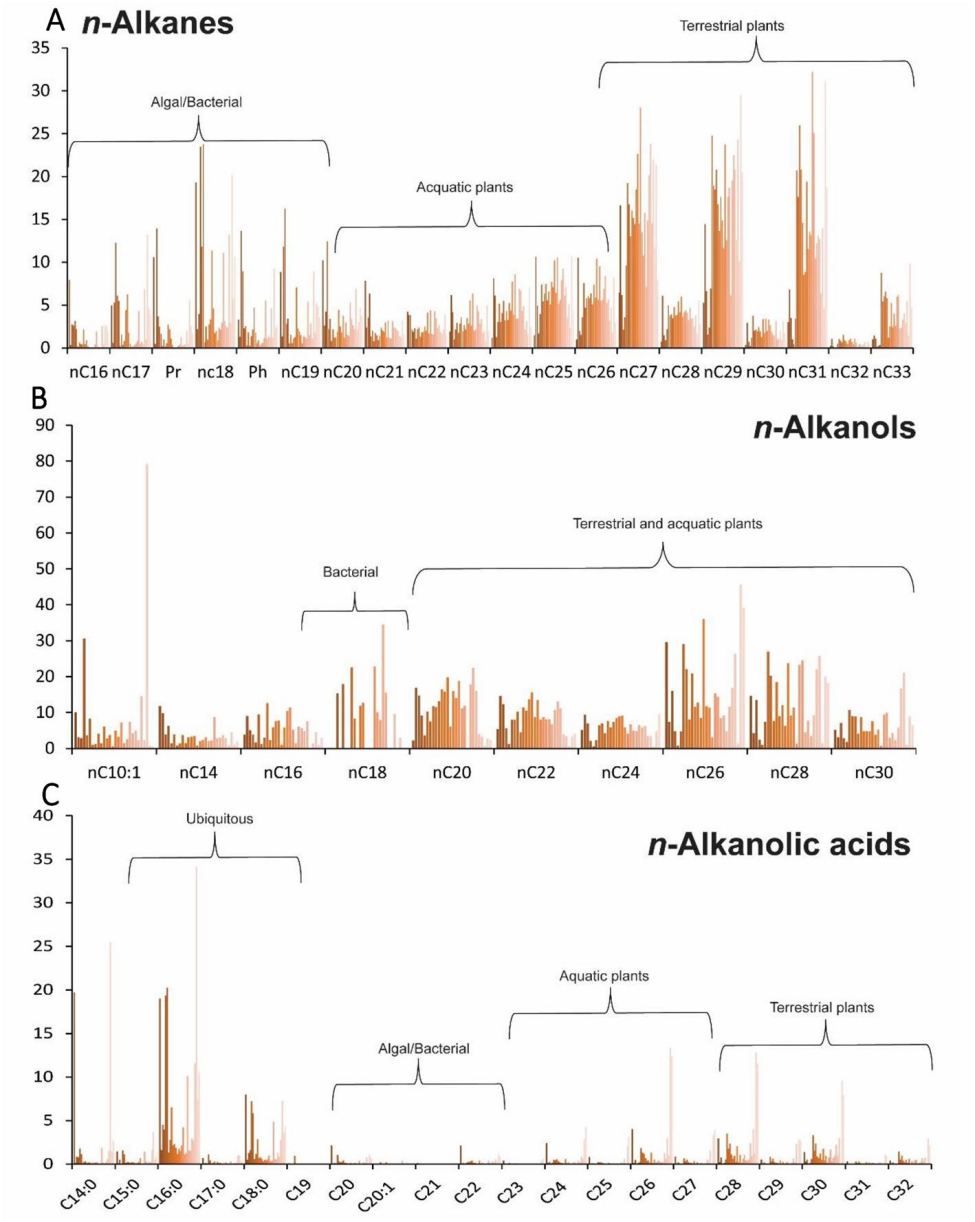
Histograms depicting the main lipids identified in the GC–MS analyses of 3 polarity fractions. (**A**), percentages of the n-alkanes; (**B**), percentages of n-alkanols; (**C**), percentages n-alkanoic acids.

**Figure 5:**
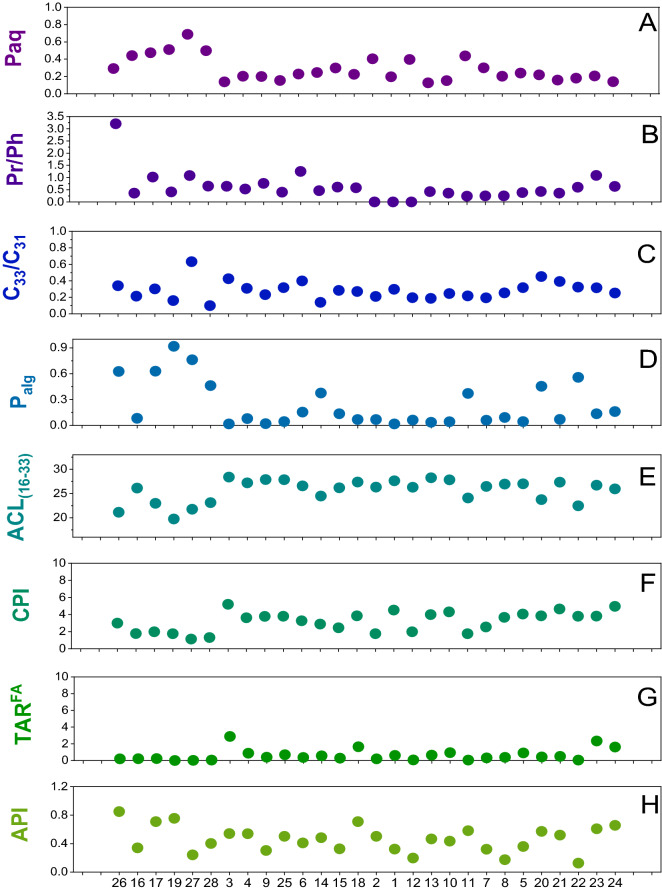
Geochemical proxies plots from Valdocarros II in the Jarama Valley. (**A**) P_aq_, ratio of macrophytic lipids (n-C23 + n-C25) relative to macrophytic and terrestrial lipids (n-C23 + n-C25 + n-C29 + n-C31) (< 0.4 = no macrophytes; 0.4 to 1 = emergent macrophytes; > 1 = floating macrophytes). (**B**) Pr/Ph, pristane to phytane (higher values more oxic conditions). (**C**) C_33_/C_31_: Ratio of n-C_33_ to n-C_31_, (higher values, more grasses). (**D**) Palg: Ratios of algal lipids (n-C_17_ + n-C_19_) relative to algal and terrestrial plant lipids (n-C_17_ + n-C_19_ + n-C_29_ + n-C_31_) higher values, more algal input). (**E**) ACL = ∑(Cn × n)/∑(Cn) Average chain length of individual n-alkane abundances. (**F**) CPI = [∑odd(C_21-33_) + ∑odd(C_23-35_)]/(2∑even C_22-34_) Carbon preference index, indicative of the abundance of odd over even carbon chain lengths (lower CPIs often indicative of microbial degradation or maturation of the sample). (**G**) TAR^FA^: Terrigenous to aquatic n-alkanoic acids ratio reflecting the importance of terrigenous and aquatic sources (C_24_ + C_26_ + C_28_)/(C_14_ + C_16_ + C_18_) (higher values, more terrestrial input). (**H**) API: alcohol preservation index uses only n-hexacosanol and n-nonacosane (higher values more hypoxic conditions).

Plant biomarkers are widely used to reconstruct the vegetation and (paleo)climate in ancient environments. This is the first application of biomarkers at the Valdocarros site in Spain. All samples from Valdocarros yielded a significant number of homologous *n*-alkanes spanning between C_16_ and C_33_ (Figs. 4A and 5). All samples show a distribution dominated by long-chain and short-chain, odd-numbered homologs (ACL: average chain length^44,45^) = 29.5) (Fig. 5E,F), indicative of mixed C3-C4 plant inputs^44–46^. The biomolecular proxy P_aq_ (ratios of macrophytes lipids relative to macrophytes and terrestrial lipids^46^, Fig. 5A) indicates the proportion of submerged and floating macrophytes versus emergent macrophytes and terrestrial plants. Samples at Valdocarros have P_aq_ aquatic index between 0.1 and 0.8 (Fig. 5A), which correspond to emergent and floating macrophytes such as from the genus *Typha*. Samples from Hearth-1 (H-1; samples #27, 28, 19, 17, 16) show the highest values corresponding to submerged macrophytes. Additionally, the P_alg_ (ratio of algal lipids [*n*C_17_ + *n*C_19_] relative to algal and terrestrial plant lipids [*n*C_17_ + *n*C_19_ + *n*C_29_ + *n*C_31_]) indicates the proportions of algal input, higher values mean more algal input. Samples from Valdocarros have a P_alg_ ratio^47^ that is lower than 1 (Fig. 5D) and indicate a low presence of algal organic matter, except samples from H-1 that show values around 0.8 indicating higher algal input. The *n*C_33_/*n*C_31_ ratio has been indicated to show changes in grass abundance (higher values, more grasses)^48,49^. Our ratio shows values ranging from 0.1 to 0.6, average values closer to 0.6 suggest less grass abundance^48^.

### Oxic/hypoxic conditions

The proportion of *n*-hexacosanol (*n*-alcohol C_26_) and *n*-nonacosane (*n*-alkane C_29_) (called alcohol preservation index [API]) has been suggested to be indicative of bottom-water oxygenation changes^50,51^. Our API values range from 0.1 to 0.8 (Fig. 5H), average values above 0.4 suggest hypoxic conditions and values below 0.2 oxic conditions^50^. Thus, we interpret that Valdocarros was an intermittently waterlogged environment, situated near a perennial river with seasonally shifting meanders across its floodplain.

Pristane and phytane derive from the phytol side-chain of chlorophyll^45,52,53^. Redox conditions influence the diagenetic pathway, promoting phytol conversion to phytane; while, oxic conditions promote the conversion of phytol to pristane^53^. Pr/Ph values of less than one (< 1) indicate anoxic deposition; in contrast, Pr/Ph values above one indicates oxic deposition. At Valdocarros, all sampled sediments have Pr/Ph ratios of 0-to-1 (Fig. 5B), besides sample #26 (charcoal) that has a value of 3.2. We interpret the overall low Pr/Ph values as corroborative molecular evidence of frequently waterlogged soil-forming conditions at Valdocarros which also underscore the intention (i.e., foresight) required to maintain continuous burning, even though soil moisture may have been paradoxically used to control the extent of the fire^54,55^.

### n-Alkanoic acids

The intermediate polarity fraction of Valdocarros extracts shows a typical bimodal distribution of mid- and long-chain (C_16:0_-C_18:0_ and *n*C_24:0_-*n*C_32:0_, respectively)* n*-alkanoic acids (Fig. 4C) with an even-over-odd predominance, that is consistent with a mix of aquatic and terrestrial C3 plant sources. Longer chain fatty acids (*n*C_26:0_-*n*C_32:0_) originated from higher plants and are relatively low in abundance. Shorter chain *n*C_14:0_,* n*C_15:0_, C_16:0,_ and C _18:0_ are produced by all plants and organisms, though are dominant in algae/aquatic plants and bacteria^56^. The terrigenous to the aquatic ratio (TAR^FA^) is the ratio between the concentration of short-chain to long-chain fatty acids and determines aquatic organic matter versus terrestrial one^57^. Higher TAR^FA^ values indicate increased terrigenous sources of lipid organic matter relative to aquatic sources. Our TAR^FA^ values (Fig. 5G) are low, demonstrating that at Valdocarros site the aquatic/algal organic matter input was predominant^57^. However, the relative abundances of C_16:0_ and C _18:0_ are very low compared to the ketones.

### Ketones

Mid-chain ketones (in the range of C_31_ to C_35_) were detected in sediments at Valdocarros. The presence of these molecules is associated with ketonic decarboxylation reactions in clay-rich environments at temperatures of 450 °C^58–61^. The presence of these compounds offers direct evidence of fat being heated to relatively high temperatures^62^. During the ketonic decarboxylation reactions two carboxylic acid functional groups are converted into a carbonyl group, plus carbon dioxide and water^61^. Interestingly, previous studies of burning pine wood have not identified ketone components in fires without bone or animal flesh^63^. Mid-chain ketones are also found in molecular residues of ‘bone fires’, in which bone itself serves as fuel^64–66^.

**Figure 6:**
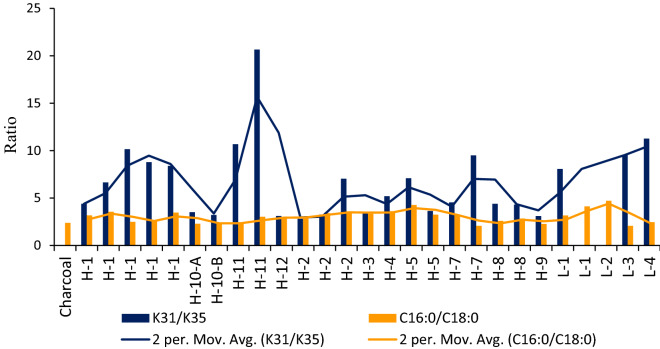
Histograms comparing the ratio of relative abundances of mid-chain ketones (K31 and K35), and free fatty acids ratio relative abundances (C16:0 = palmitic acids and C18:0 = stearic acid).

Condensation of two C_16:0_ fatty acids forms a C31 ketone (K_31_), and the condensation of two C_18:0_ fatty acids forms a ketone with 35 carbons (K_35_)^62^. Figure 6 shows that in all cases the C_16:0_/C_18:0_ ratio is lower than the K_31_/K_35_ ratio; thus, demonstrating that the high temperatures were reached by most samples from Valdocarros II, because the higher the temperatures the longer chains ketones are formed by the ketonic decarboxylation reactions. These suggest that at Valdocarros II heating up sediments has occurred. The sediments showed reddening and darkening reaching up to a few centimetres in depth. This characteristic has been recorded from multiple studies in paleo-fire and laboratory experiments^63,64^. Further molecular evidence of fire at Valdocarros—such as shorter-chain diacids viz. methyl 9,10-dihydroxystearate and undecanedioic acid—are also indicators of bone combustion^64,67,68^. Samples from Hearth-11 (#9 and #25) showed the highest K_31_/K_35_ ratio (Fig. 6), probably indicating that Hearth-11 was perhaps used for cooking meat. Samples from Hearth-7 showed a slightly high K_31_/K_35_ ratio compared to C_16:0_/C_18:0_ ratio.

### Alkylated polycyclic aromatic hydrocarbons

**Figure 7:**
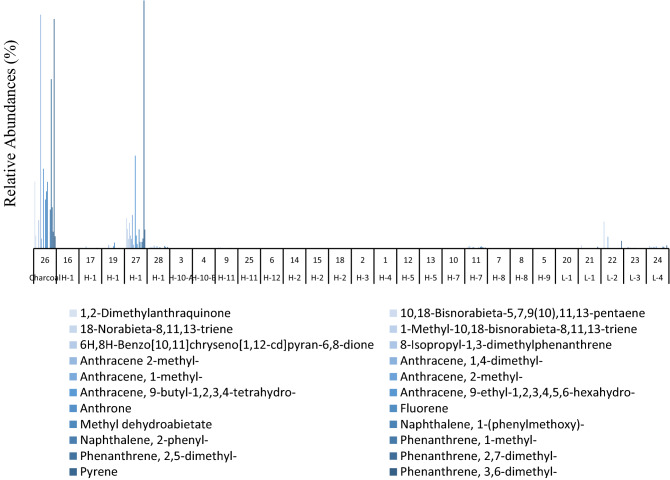
Histograms comparing the relative abundances of PAHs and APAHs from samples collected at Valdocarros II in Jarama Basin.

**Figure 8:**
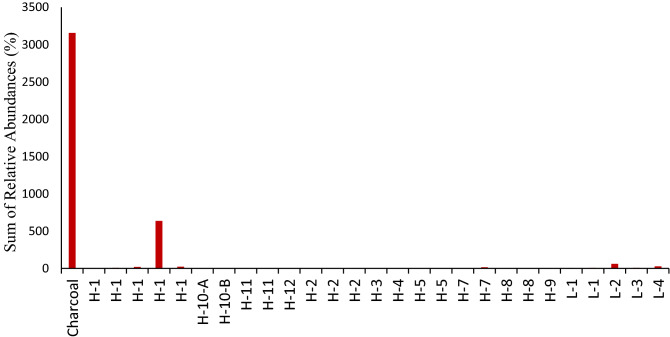
Histograms comparing the sum of APAHs from samples collected at Valdocarros II in Jarama Basin.

Evidence of the presence of polycyclic aromatic hydrocarbons (PAHs) and alkylated PAHs (APAHs) have been previously found in archaeological sediments samples associated with burning activities^69^. PAHs and APAHs were detected in different abundances in most samples from Valdocarros II (Fig. 7). The most prominent and ubiquitous source of PAHs and APAHs is the incomplete combustion of biomass (such as wood and bones)^70^. Most samples revealed abundances of 3-ring, 4-ring PAHs and 2-ring, 3-ring methyl-, and di-methyl APAHs that are indicators of wood-burning (Fig. 8). Di- and methyl-phenanthrenes, di- and methyl-anthracenes, phenyl-naphthalene, pyrene, and fluorene were detected. All these compounds are indicative of the combustion of organic matter and their co-occurrence is an indicator of pyrolytic processes. Anthracene and phenanthrene were not detected in any samples, probably because of the incomplete combustion of wood and bones^71^. APAHs were more abundant than PAHs which is indicative of a low-temperature fire^71^ and it is probably an indicator of incomplete burning due to temperatures not exceeding 350–500 °C, as well the 3-ring APHAs are most abundant, which are characteristics of low-temperature fire that burn around 100–150 °C^71^. The abundances of PAHs and APAHs differ between samples due to the heterogenic character of samples and location, samples from Hearth-1 and sample #26 show the highest abundances of both APAHs and PAHs. Our evidence from Hearth-1 and samples #26 show that burning activities were carried out by hominins at specific locations. Background sediments (L-1, L-2, L-3, L-4) do not show the presence of either APAHs or PAHs (Figs. 7 and 8).

### Terpenes

**Figure 9:**
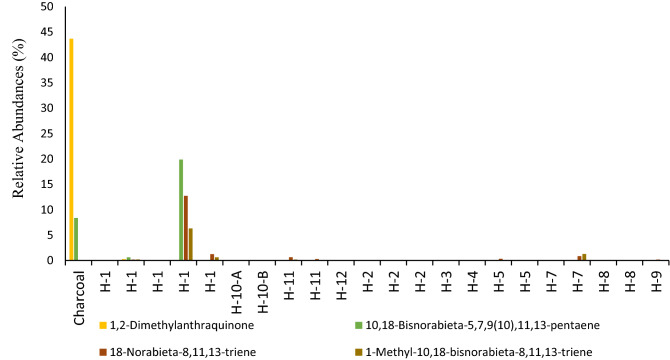
Histograms comparing the relative abundances of triterpenes from samples collected at Valdocarros II in Jarama Basin.

**Figure 10:**
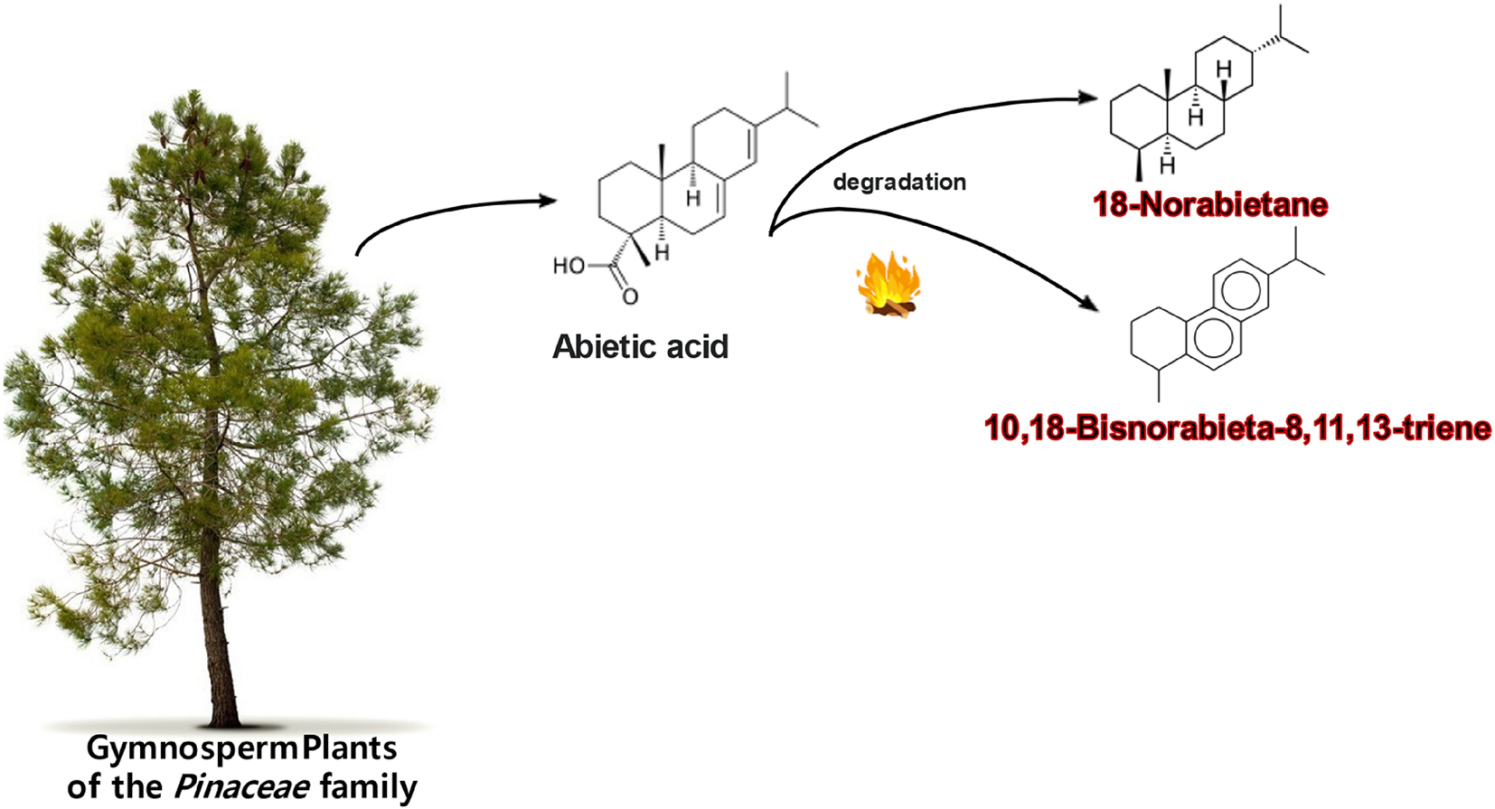
Summary of degradation pathway of abietic acid produced by gymnosperm plants. This figure was made by Lavinia Stancampiano.

Samples from Hearth-1 and sample #26 showed presence of 18-norabieta-8,11,13-triene, 10,18-bisnorabieta-5,7,9(10),11,13-pentaene that are degradation products of diterpenoid acids that indicate conifers wood burning (Figs. 9, 10)^72,73^. Only samples from Hearth-1 and sample #26 recorded high abundances of 10,18-Bisnorabieta-5,7,9(10),11,13-pentaene, and background sediments showed very low abundances of such compounds. This evidence is supporting that at Valdocarros II Hearth-1 early hominins were heating conifer wood. Friedelan-3-one has also been identified in all samples, and it is characteristic of higher plants^74,75^. The presence of di-hydroxy-hexadecanoic acid isomers (such as 16-hydroxy-hexadecanoate) is a major component in conifer needles, and it has been identified in many samples from Valdocarros II^76,77^. Our results suggest that probably hominins were burning conifer wood at Valdocarros II.

Moreover, the co-occurrence of saturated *n*-acids longer than C_18_, *n-*alkanols longer than C_17_, di-acids, dihydroxy acids, long chains ketones, *n*-alkanes, norabietanes, and APAHs and PAHs demonstrate that at Valdocarros II there were human-controlled fires, and that in particular Hearth-1 was a human-controlled fire probably made by the combination of conifer wood and bones/meat.

### Alcohols

We have found ergosta-5,22-dien-3-ol and ergosta-7,22-dien-3-ol in all samples from Valdocarros II. Previous studies identified such compounds in wood-decay (xylophagous) fungus^78–80^. The highest abundances were found in samples #28 (from Hearth-1) and sample #9 (from Hearth-11), suggesting general fungal presence at Valdocarros with localized intensification at the largest hearths. These results suggest that early hominins at Valdocarros were burning pre-fallen and fungi-rotted—as opposed to fresh-cut—tree material. In turn, the use of deadwood by hominins offers unique insights into foraging practices for resource selection and landscape utilization, which is otherwise ‘invisible’.

The analysis of the polar lipid fraction clarifies the paleoenvironmental conditions at Valdocarros II site. The major *n*-alcohols present in the lipid extracts are long-chain *n*-alkanols ranging from C_24_ to C_32_ and show a very strong predominance of even-carbon chain-lengths (Fig. 4B). Longer even-numbered C_24,26,28_ and C_30_ chain lengths are typical of aquatic and terrestrial inputs (Fig. 4B), and C_24_ *n*-alcohol have been found in freshwater phytoplankton^81,82^. Microalgae produce long-chain alcohols, C_26_ and C_28_ *n-*alcohols are produced by freshwater *Eustigmatophyceae*^82^. Our evidence from Valdocarros II shows a strong aquatic organic matter input that is supported by the high proportion of long chains *n*-alkanols that are typically produced by algae. This is following the geological and sedimentological data that indicates an abandoned meander^36^. Moreover, studies of the herpetofauna^43^, micromammals^83^ recorded the presence of *Castor fiber* and *Arvicola aff*. *Sapidus*^84,85^ indicates the necessary presence of flowing water and humidity. Our biomarkers analyses, combined with previous studies^42,43,83^, can infer that the environment at Valdocarros II was probably surrounded by woodland and riverside vegetation, probably few shrubs and few grasses.

## Evolutionary perspectives on controlled fire

The use of fire, in conjunction with stone tool manufacture, is one of the most important developments in all human evolution^11,12,86^. With this in mind, fire management implies multiple cognitive and behavioral faculties: intensive conceptual knowledge of the environment (e.g., to obtain relevant fuel), prediction of fire requirements (e.g., placement), the ability to (re)ignite combustion, and the existence of basic economic cooperation via social interaction, as obtainment of fuel has an energetic cost^87–89^.

Before MIS 13 (ca. 528 ka), evidence of fire-use is indirect (for instance, interpreting patterns in charcoal fragment dispersal at Sima del Elefante level TE19 G^27^; heat-altered bone and flakes at Cueva Negra del Rio Quípar^28–30^) and features no indication of long-term fires or repeat hearth use. In Europe, some authors suggested that between MIS 13 and MIS 9 (ca. 528–334 ka) human-controlled fire-use was common across the Iberian region^11,12^. Barsky^13^ further suggest that controlled fire-use was associated with the regional expansion of the Acheulean technocomplex, ca. 500–600 kya, although the majority of Acheulean archaeological sites show no strong evidence of hearths.

Within this framework, there were clustered burnt bones at Vérteszöllöos (Hungary) in a level with proto-handaxes^14^, though James et al*.*^6^ considered that thermal features vis-a-vis mineral stains could be due to climate-induced diagenesis; fireplaces associated with charcoals and burnt tools at Menez-Dregan 1 (France), a marine cave, dated ca. 465 and 380 ka by ESR, with handaxes at least in level 7^15,16^; burnt chert at La Grande Vallée (France)^17^, ca. 350 ka, together with the production of handaxes (unit 5); accumulations of burnt remains forming semi-circular areas at Bilzingsleben (Germany), dated between 350–320 ka and 414–280 ka^19^, but no evidence of fire was produced by human^89^, and a level with knives, backed-knives called Keilmesser, and *handaxe shaped points*; possible hearths, burned sediments and wooden at Schöningen (Germany)^70^, though recently Stahlschmidt et al. (2015) carried out several analysis and they considered that there were not solid evidences of human use of fire and that the stone tools recalled the lithic assemblage of Bilzingsleben with heavy-duty small scrapers; at Terra Amata (France) charcoal and burnt material^18^ with a *“credible evidence of fire”* according to^22^ several combustion structures^90^ and the chronology of the site, was placed between 250 and 400, but TL dated to 230 ka, and the mammal assemblage situates it between MIS 9 or 11^91^ and the lithic industry is characterized by choppers, picks, handaxes and cleavers made on pebbles but no on large flakes; burnt flint, bone and thermally altered sediments interpreted as remains of hearths at La Beeches Pit, England^20^ dated by TL,U-series and AAR around 400 ka, but by OSL at 200 ka^92^, and with handaxes also have been found; products of burning, composed by bone, charcoal and possibly quartzite cobbles at Gruta da Aroeira, Portugal^21^, ca. 400 ka, with bifaces on flake but no cleavers on flake^93^.

### Characteristics of human-controlled fire

Temperature is a reliable criterion to discriminate wildfires and bonfires or campfires (N.B., larger and smaller controlled fires, respectively) since early Pleistocene hominins were almost certainly not making fires with temperatures above 800 °C^86,94^. Typically, a wildfire catches quickly and reaches comparatively high temperatures as compared to controlled fires (i.e., > 800 °C versus < 450 °C, respectively) and spreads quickly throughout the landscape^95^. On the other hand, bonfires, which are usually composed of wood and bone^64^, are contained to a specific, restricted location with fire and heat in the same location. With this in mind, the spatial distribution of features at Hearth-1 at Valdocarros II looks consistent with a controlled (bon)fire rather than at wildfire (Fig. 2A). Hearth-1 is about 3 m wide with crisscrossed blackened fragments organized in a circle organization with dark red sediment in its centre, possibly a concentrated focus of heat^63,64^. Indeed, the structure of Hearth-1 is uniquely indicative of human-controlled fire^63^. Bones and tools from Valdocarros II do show thermal alteration in the two levels (manuscript *In Review*), which might indicate early hominins did not use such objects around the fire itself.

### Potential hominin uses of fire at Valdocarros II

Valdocarros II lies in an abandoned meander, with samples at Hearth-1 featuring proportional highest inputs of freshwater organic matter, corresponding to deeper water depth while the meander was still active before hominins occupation. This suggests that hominins have preferentially chosen the deeper parts of the abandoned meander as it would have provided the highest shelter from wind and other predators. The controlled use of fire has been associated as a source of heat and source protection against predators also at Koobi Fora FxJj 20 Main site^96^.

Valdocarros II Hearth-1 looks much like a structured bonfire, with evidence of a defined ~ 10 m^2^ hearth formed by pinewood arranged in a circle and likely smaller hearths located in the area to protect hominins from possible predators^97^. For instance, felids (hyenas and foxes) are scared by fire and hominins could have used fire to keep them at away^35,98,99^. The hearths at Valdocarros II were probably used such as for defence against external threats, as inferred^97^ the use of fire by humans represents their predominance over other mammals. Also, the indicators of bone burning can explain how the fire could be used as a toxin neutralizer. The roasting can have a preservative effect to minimize the oral digestion of bacterial and parasitic load in meat, increase the digestibility and the absorption of nutrients^100,101^.

Biomarkers from Valdocarros build upon earlier reconstructions of the Jarama Basin^33,41–43^ suggesting (paleo)environmental and climatic conditions exerted a direct influence on regional hominin behaviours. Valdocarros II itself harboured occasional large woody plants, such as conifers, and based on pollen spectra, most trees occurred on river edges^42^. Further, existing pollen spectra at Valdocarros suggest Mediterranean woodland taxa (dominated by *Pinus* with nominal *Cupressaceae*) typified the vicinal landscape with interspersed habitats rich in aquatic riverside vegetation^42^. Biomarkers across Valdocarros II also show distribution characteristics of aquatic plants such as freshwater algal, floating, and submerged macrophytes, that would not be depicted otherwise. Our data suggest that critical resources had a direct implication on hominin behaviours. The occurrence of district plants, or otherwise, unvegetated fluvial-meandering environment, which integrates a vicinal river organic matter input dominated by macrophytes.

Valdocarros II was dominated by aquatic plants, few trees, and shrubs, and probably early hominins were occupying the site also for its proximity to flowing water where to obtain biotic and abiotic resources. Our data indicate that hominins took strategic advantage of a largely vegetated location in addition to caves in the Iberian Peninsula, suggesting that hominins had a strategic understanding of environment and space. This is the first open-air site in the Iberian Peninsula that shows evidence of the control of fire despite the possibility of flooding and weathering that could have eroded such evidence. Especially the high abundances of bis-norabietanes indicate that hominins were burning wood collected from the nearby woodlands, which were dominated by *Pinus*^42^.

Valdocarros archaeological site is one of the largest excavated Acheulean sites, together with Torralba and Ambrona sites (Soria) in the Iberian Peninsula and show numerous assemblages of faunal remains and Acheulean artifacts in five levels. This suggests that area must have been occupied for repeated episodes for carcass consumption on a possible bonfire.

## Conclusions

Our interdisciplinary, multi-proxy analyses of combustion (burning) by-products suggest the presence of anthropogenic (controlled) fires at Valdocarros II. This site is one of the few Middle Pleistocene examples of anthropogenic fire recorded in Europe to date; with a clear chronological framework, is one of the oldest pieces of evidence of fire-use at open-air Acheulean site in Europe together with Terra Amata in France and the only one in the Iberian Peninsula. The burnt material of Valdocarros II consists of wood and charcoal, high concentration, and diversified PAHs alongside diagnostic conifer-derived triterpenoids. The two hearths presented high abundances of alkylated 3-ring PAHs and norabietane derivatives suggesting that the two hearths were anthropogenic fires and conifer wood was used as fuel.

These finds were made in an abandoned meander and in two contiguous levels in association with bones and the Acheulean lithic industry. The results, scarce to date, add new data to the knowledge of the use of fire by hominins during the Middle Pleistocene in Europe, and they are an approach to the complex mind of Acheulean groups and their interactions with the Pleistocene environment.

## Materials and methods

### Sampling

Excavation and sample collection were carried out following geologic stratification (lithostratigraphy) at Valdocarros II. Representative sediment samples (~ 50 g; *n* = 28) were collected with the use of a metal spoon. Sample lists and locations are reported in Table 1 and Fig. 3A,B. All glassware used was combusted at 450 °C for 6 h.

**Table 1:** Samples list and fluvial unit locations.

Fire hearths loc	Sed layer	Sample #
*H-1*	1	27
*H-1*	1	28
*H-1*	1	19
*H-1*	1	17
*H-1*	1	16
*H-2*	2	18
*H-2*	2	14
*H-2*	2	15
*H-3*	1	02
*H-4*		01
*H-5*	1	12
*H-5*	1	13
*H-7*	1	10
*H-7*	1	11
*H-8*	1	07
*H-8*	1	08
*H-9*	1	05
*H-10-A*	1	03
*H-10-B*	1	04
*H-11*	1	09
*H-11*	1	25
*H-12*	1	06
*Charcoal*	1	26
*Sediments*	1	20
	1	21
	2	22
	3	23
	4	24

### Biomarker extraction and isolation

Sediment samples were freeze-dried and powdered with a solvent-clean agate mortar and pestle. Extraction was performed using an accelerated solvent extractor (Dionex ASE 350 system) with dichloromethane (DCM) and methanol (MeOH) (4:1 vol/vol) in 3 cycles at 100 °C (10.3 MPa) with a static time of 5 min^102^. The resultant total lipid extract (TLE) was dried under a gentle stream of nitrogen and then derivatized via acid methanolysis (0.5 M HCl in methanol [MeOH] diluted with ultra clean water (milli-q water washed 3 times with DCM) before subsequent liquid–liquid isolation into hexane: DCM (4:1 vol/vol)^47^. Derivatized TLEs were concentrated and chromatographically partitioned into three fractions using deactivated silica gel^103^ (2% H_2_O total weight) by elution with hexane (F1), hexane: DCM (1:1 [F2]), and DCM: MeOH (4:1 [F3])^47^. Polar (F3) fractions were silylated using N,O-bis(trimethylsilyl) trifluoroacetamide (BSTFA).

### Molecular analysis

Biomarkers were identified by Gas Chromatography-Mass Spectrometer (GC–MS; model Thermo Scientific™ TRACE™ 1310 [GC] with coupled ISQ LT [MS]) by injecting in splitless mode 1 mL aliquot of apolar and derivatized fractions onto a 60 m-VF1 column fused-silica column (0.25 mm × 0.25 mm). The GC oven was programmed as follow: 60 °C injection and hold for 2 min, ramp at 10 °C min to 150 °C, ramp at 4 °C min to 300 °C followed by isothermal hold of 20 min. The transfer line and source are set at 320 °C and 270 °C respectively. Procedural blanks were run to monitor background interferences. Data were acquired and processed under the conditions described and each sample was run in duplicates. Compound identifications were made via comparison with authentic standards (QTM PAH Mix Supelco, Supelco 37 component FAME mix, and from Mixture B4 [Schimmelmann Standards]) in conjunction with the NIST 20 electron ionization spectral library.

## Supplementary Information

Supplementary Information.

## Data Availability

All data generated or analysed during this study are included in this published article or in the accompanying Supplementary Information file.
